# Supplementary material for: Transcriptomic and Proteomic Analysis Reveals Mechanisms of Patulin-Induced Cell Toxicity in Human Embryonic Kidney Cells
Source: Toxins (Basel). 2020 Oct 29;12(11):681. doi: 10.3390/toxins12110681 (PMC7692636; doi:10.3390/toxins12110681)
Supplement: Supplementary file 1 [file toxins-12-00681-s001.pdf]

Supplementary Materials: Transcriptomic and Proteomic Analysis Reveals Mechanisms of Patulin-Induced Cell Toxicity in Human Embryonic Kidney Cells

Nianfa Han, Ruilin Luo, Jiayu Liu, Tianmin Guo, Jiayu Feng and Xiaoli Peng

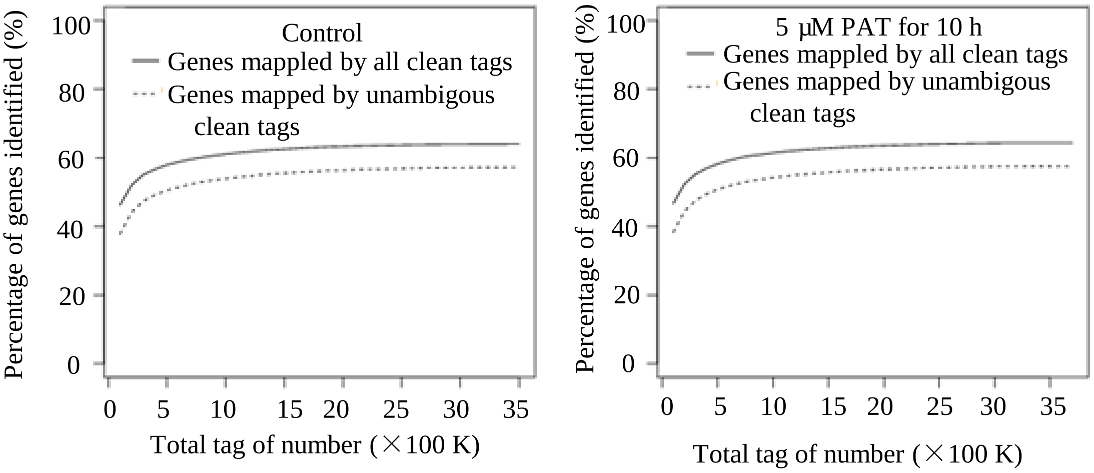

Figure S1. Saturation evaluation of detected gene expression. When sequencing amount reaches 2 M or higher, the number of detected genes almost ceases to increase.

Table S1. Statistics of categorization and abundance of digital gene expression (DGE) tags.

	Control				5 μM PAT for 10 h			
	**Total tags**		**Distinct tags**		**Total tags**		**Distinct tags**	
	number	%	number	%	number	%	number	%
Total reads	3,479,005	100.00	335,487	100.00	3,732,185	100.00	359,859	100.00
Tags containing N	3309	0.10	2757	0.82	3328	0.09	2760	0.77
Only adaptors	32	0.00	27	0.01	91	0.00	86	0.02
Copy number < 2	193,285	5.56	193,285	57.61	210,396	5.64	210,396	58.47
clean reads	3,282,379	94.35	139,418	41.56	3,518,370	94.27	146,617	40.74
Copy number (2,5)	238,686	7.27	83,877	60.16	248,991	7.08	87,676	59.80
Copy number (6,10)	160,649	4.89	21,200	15.21	169,270	4.81	22,339	15.24
Copy number (11,20)	208,339	6.35	14,231	10.21	219,891	6.25	15,076	10.28
Copy number (21,50)	351,955	10.72	11,130	7.98	372,749	10.59	11,823	8.06
Copy number (51,100)	314,553	9.58	4479	3.21	339,517	9.65	4827	3.29
Copy number > 100	2,008,189	61.18	4501	3.23	2,167,952	61.62	4876	3.33
PM (sense) 1 tag > 1 gene	2,188,742	66.68	69,056	49.53	2,421,411	68.82	71,975	49.09
PM (sense) 1 tag > n gene	249,016	7.59	2795	2.00	241,078	6.85	2881	1.96
1MM(sense) 1 tag > 1 gene	43,189	1.32	4749	3.41	41,443	1.18	4742	3.23
1MM(sense) 1 tag > n gene	43,761	1.33	3438	2.47	30,142	0.86	3682	2.51
PM(antisense)1 tag > 1 gene	248,745	7.58	23,290	16.71	273,482	7.77	25,157	17.16
PM(antisense)1 tag > n gene	18,954	0.58	825	0.59	19,985	0.57	846	0.58
1MM(antisense) 1 tag > 1 gene	6659	0.20	1016	0.73	5549	0.16	943	0.64
1MM(antisense) 1 tag > n gene	938	0.03	146	0.10	909	0.03	146	0.10
PM genome 1 tag > 1 position	141,139	4.30	20,460	14.68	151,996	4.32	22,219	15.15
PM genome 1 tag > n position	61,570	1.88	582	0.42	63,519	1.81	588	0.40
1MM genome 1 tag > 1 position	17,776	0.54	1509	1.08	15,898	0.45	1451	0.99
1MM genome 1 tag > n position	7587	0.23	826	0.59	6676	0.19	753	0.51
Unknown tag	254,303	7.75	10,726	7.69	246,282	7.00	11,234	7.66

The total tag number and the distinct tag number statistics of the control and 5 μM PAT treated for 10 h in HEK293 cells. Raw sequences have 3′ adaptor fragments as well as a few low-quality sequences and several types of impurities. Raw sequences are transformed into clean tags after certain steps of data-processing. Total tags represent the sum of all tag number, while distinct tags represent all types of tags. "Tags containing N" means tags with unknown sequences 'N'. "Only adaptors" means the reads contain only the adaptors sequence. "Copy number < 2" is the tags whose copy number is less than 2. "Clean tags" is the tags used to analysis after filtering the dirty tags. Copy number [x, y] means the copy number of clean tags between x and y. PM means perfect match to gene, and MM means match to gene with 1 bp mismatch. 1 tag > 1 gene and 1 tag > n gene means match to one or more than one gene respectively. 1 tag > 1 position and 1 tag > n position means match to genome sequence with one best hit or multiple best hits, respectively. Unknown tag means most match to gene (sense and antisense) and genome sequence.

Table S2. List of differentially expressed genes (twofold changes or greater, $P < 0.05$) by DEG from the human embryonic kidney cells following 5 μM PAT treatment for 10 h.

Gene symbol	Gene Name	Fold Change (log2)	transcriptID
HSPB8	Heat shock protein beta-8	739 (9.53)	NM_014365
PHF21B	PHD finger protein 21B	483 (8.91)	NM_001135862
KAZALD1	kazal-type serine protease inhibitor domain-containing protein 1 precursor	455 (8.83)	NM_030929
AGO2	protein argonaute-2	6.34 (2.67)	NM_001164623
UBASH3B	Ubiquitin associated and SH3 domain containing B	5.80 (2.54)	NM_032873
ULBP1	UL16 binding protein 1	4.66 (2.22)	NM_025218
COPRS	Coordinator of PRMT5 and differentiation stimulator	4.59 (2.20)	NM_018405
PSENEN	gamma-secretase subunit PEN-2	4.26 (2.09)	NM_172341
RBM38	RNA-binding protein 38 isoform a	4.07 (2.02)	NM_183425
BCL2L12	bcl-2-like protein 12 isoform 1	3.86 (1.95)	NM_001040668
RPS9	Ribosomal protein S9	3.73 (1.90)	NM_001013
ARL8A	ADP-ribosylation factor-like protein 8A	3.73 (1.90)	NM_138795
GNG11	Guanine nucleotide-binding protein G subunit gamma-11	3.63 (1.86)	NM_004126
CSTF3-AS1	CSTF3 antisense RNA1	3.31 (1.73)	NR_034027
MIF4GD	MIF4G domain-containing protein	3.28 (1.71)	NM_020679
HMOX1	heme oxygenase 1	3.20 (1.68)	NM_002133
UNKL	Unkempt-like RING finger protein	3.02 (1.60)	NM_023076
AJUBA	AjubaLIM protein	3.02 (1.59)	NM_198086
ST3GAL4	ST3-beta-galactosamide-alpha-2,3-sialyltransferase 4	3.02 (1.59)	NM_006278
LSM1	U6 snRNA-associated Sm-like protein LSm1	3.01 (1.59)	NM_014462
NOTCH3	Neurogenic locus notch homolog protein 3	2.92 (1.55)	NM_000435
FIS1	Fission 1	2.91 (1.54)	NM_016068
ZFP62	Zinc finger protein 62 homolog	2.87 (1.52)	NM_001172638
ARID3A	AT rich interactive domain 3	2.80 (1.48)	NM_005224
ACADS	Acyl-CoA dehydrogenase	2.80 (1.48)	NM_000017
GDPD1	Glycerophosphodiester phosphodiesterase domain-containing 1	2.75 (1.46)	NM_001165993
FOXO4	Forkhead box protein O4	2.71 (1.44)	NM_005938
RIPPLY3	Protein ripply3	2.68 (1.42)	NM_018962
GUCY1B3	Guanylate cyclase soluble subunit beta	2.67 (1.42)	NM_000857
CIAPIN1	Cytokin induced apoptosis inhibitor 1	2.63 (1.40)	NM_020313
UFC1	Ubiquitin-fold modifierconjugating Enzyme 1	2.59 (1.37)	NM_016406
HSPA8	Heat shock cognate 71 kDa protein	2.58 (1.37)	NM_006597
STIP	STAT3-interacting protein	2.58 (1.37)	NM_001037163
RNF34	E3 ubiquitin-protein ligase RNF34	2.58 (1.37)	NM_025126
PDE12	2′,5′-phosphodiesterase 12	2.57 (1.36)	NM_177966
IFI27L1	Interferon alpha inducible protein 27 like 1	2.56 (1.36)	NM_145249
MFSD5	Major facilitator superfamily domain-containing protein 5	2.55 (1.35)	NM_032889
GDI1	Guanosine diphosphate (GDP) dissociation inhibitor 1	2.51 (1.33)	NM_001493
RBM10	RNA binding motif protein 10	2.50 (1.32)	NM_152856
TM2D3	TM2 domain-containing protein 3	2.49 (1.31)	NM_078474
UQCR11	Cytochrome b-c1 complex subunit X1	2.47 (1.30)	NM_006830
NDUFA4	NADH dehydrogenase [ubiquinone] 1 alpha subcomplex subunit 4	2.45 (1.30)	NM_002489
FSCN1	FSCN1 protein	2.44 (1.29)	NM_003088
UIMC1	Ubiquitin interaction motif containing 1	2.44 (1.29)	NM_001199298
DNAJC16	DnaJ heat shock protein family member C16	2.42 (1.272)	NM_015291
BLVRB	Biliverdin reductase	2.37 (1.24)	NM_000713
FAM107B	Family with sequence similarity 107 member B	2.35 (1.23)	NM_031453
SCARB1	Scavenger receptor class B member 1	2.34 (1.23)	NM_005505
PITRM1	Presequence protease	2.34 (1.22)	NM_001242309
REEP4	Receptor expression-enhancing protein 4	2.31 (1.21)	NM_025232
C6orf136	Chromosome 6 open reading frame 136	2.29 (1.20)	NM_145029
BRCA1	BRCA1 interacting protein C-termianl helicase 1	2.29 (1.20)	NM_032043

PUM1	PumilioRNA binding family member 1	2.28 (1.19)	NM_001020658
SNX7	Sorting nexin-7	2.27 (1.18)	NM_152238
INF2	Inverted formin-2	2.27 (1.18)	NM_032714
PDXDC1	Pyridoxal dependent decarboxylase domain containing 1	2.27 (1.18)	NM_015027
SLC37A3	Solute carrier family 10 member 3	2.26 (1.18)	NM_207113
CHCHD2	Coiled-coil-helix-coiled-coil-helix domain-containing protein 2	2.26 (1.17)	NM_016139
DENND4B	DENN domain containing 4B	2.24 (1.16)	NM_014856
SLC10A3	Solute carrier family 10 member 3	2.23 (1.16)	NM_019848
PNMAL1	Paraneoplastic Ma antigen family member 8A	2.22 (1.15)	NM_018215
FIP1L1	Factor interacting with PAPOLA and CPSF1	2.21 (1.14)	NM_001134937
LINC01623	Long intergenic non-protein coding RNA 1623	2.16 (1.11)	NR_033379
PLAT	Tissue-type plasminogen activator	2.16 (1.11)	NM_000930
TMEM131	Transmembrane protein 131	2.15 (1.10)	NM_015348
C14orf166	Homeoboxprox 1	2.15 (1.10)	NM_016039
FAM135A	Family with sequence similarity 135 member A	2.15 (1.10)	NM_001162529
POLR1C	DNA-directed RNA polymerases I and III subunit RPAC1	2.14 (1.09)	NM_203290
AIF1L	Allograft inflammatory factor 1-like	1.13 (1.09)	NM_001185096
POMGNT2	O-link mannose N-acetyglucosaminyltransferase 2	2.12 (1.08)	NM_032806
NCDN	Neurochondrin	2.12 (1.08)	NM_014284
METTL9	Methyltransferase-like protein 9	2.11 (1.08)	NM_001077180
MRPS2	Mitochondrial ribosomal protein S2	2.11 (1.08)	NM_016034
RNF181	E3 ubiquitin-protein ligase RNF181	2.11 (1.08)	NM_016494
DNAJA3	DnaJheat shock protein family (HSP40) member A3	2.11 (1.08)	NM_001135110
HMGN1	High-mobility group nucleosome binding domain 1	2.09 (1.06)	NM_004965
HSD17B10	Hydroxysteroid 17-beta dehydrogenase 10	2.08 (1.06)	NM_004493
SLC7A5	Solute carrier family 7, member 5	2.07 (1.05)	NM_003486
RPS20	40S ribosomal protein S20	2.07 (1.05)	NM_001146227
HDDC2	HD domain containing 2	2.03 (1.02)	NM_016063
RPLP2	Ribosomal protein lateral stalk subunit P2	2.02 (1.02)	NM_001004
YAF2	YY1 associated factor 2	2.02 (1.02)	NM_005748
IGBP1	Immunoglobulin-binding protein 1	2.02 (1.01)	NM_001551
MIR567	Micro RNA 567	0.15 (−2.80)	NR_030292
NAA20	N-alpha-acetyltransferase 20	0.29 (−1.80)	NM_181527
RNF148	Ring finger protein 148	0.31 (−1.69)	NM_198085
CA7	Carbonic anhydrase 7	0.32 (−1.66)	NM_001014435
CAPRIN2	Caprin-2	0.32 (−1.66)	NM_032156
HTR1D	5-hydroxytryptamine receptor 1D	0.32 (−1.64)	NM_000864
CENPS	Centromere proteins S	0.33 (−1.61)	NM_199294
ATP6V1C2	ATPaseH+V- transporting V1 subunit C 2	0.34 (−1.57)	NM_001039362
FAM173A	Family with sequence similarity 173 member A	0.35 (−1.53)	NM_023933
STAB1	Stabilin 1	0.36 (−1.48)	NM_015136
HYPM	Huntingtin interacting protein M	0.37 (−1.45)	NM_012274
LYG2	lysozyme g-like protein 2	0.37 (−1.44)	NM_175735
PI4KA	Phosphatidylinositol 4-kinase alpha	0.38 (−1.38)	NM_058004
RPL11	Ribosomal protein L11	0.39 (−1.35)	NM_001199802
NINJ1	Ninjurin-1	0.40 (−1.31)	NM_004148
MZF1AS1	MZF1 anti sense RNA1	0.42 (−1.27)	NR_027334
C16orf59	Chromosome 16 open reading frame 59	0.42 (−1.26)	NM_025108
SNHG8	Small nucleolar RNA host gene 8	0.42 (−1.26)	NR_034011
SNAP47	Synaptosomal-associated protein 47	0.42 (−1.26)	NM_053052
IRS4	Insulin receptor substrate 4	0.42 (−1.25)	NM_003604
RHOC	Ras homolog gene family member C	0.42 (−1.25)	NM_001042678
CASC2	Cancer susceptibility 2	0.44 (−1.17)	NR_026940
MZF1	Myeloid zinc finger 1	0.45 (−1.17)	NM_198055

ASMT	Acetylserotonin O-methyltransferase	0.45 (−1.16)	NM_0011710<br>38
URB1	Nucleolar pre-ribosomal biogenesis protein 1	0.45 (−1.16)	NM_014825
ANKRD6	Ankyrin repeat domain-containing protein 6	0.45 (−1.15)	NM_014942
AMBRA1	Activating molecule in BECN1-regulated autophagy protein 1	0.45 (−1.14)	NM_017749
CDKN2D	cyclin-dependent kinase 4 inhibitor D	0.46 (−1.14)	NM_079421
TBC1D17	TBC1 domain family member 17	0.46 (−1.12)	NM_0011682<br>22
DENND3	DENN domain containing 3	0.46 (−1.12)	NM_014957
POLM	DNA-directed DNA/RNA polymerase mu	0.46 (−1.11)	NM_013284
PKMYT1	Membrane-associated tyrosine/threonine 1	0.47 (−1.10)	NM_182687
SHB	SH2 domain containing adaptor protein B	0.47 (−1.09)	NM_003028
AATF	Apoptosis antagonizing transcription factor	0.48 (−1.06)	NM_012138
ZNF79	Zinc finger protein 79	0.48 (−1.06)	NM_007135
BTF3L4	BTF3L4 protein	0.48 (−1.05)	NM_024350
YBX3	Y-box binding protein 3	0.48 (−1.05)	NM_003651
SERF2	Small EDRK-rich factor 2	0.48 (−1.05)	NM_0010181<br>08
TSPAN19	Tetraspanin 19	0.49 (−1.02)	NM_0011009<br>17
POP7	POP7 homology, ribonuclease P/MRP subunit	0.50 (−1.01)	NM_005837
SNAR-E	Small ILF3/NF90 associated RNA E	0.50 (−1.01)	NR_024258
CPSF4	cleavage and polyadenylation specificity factor subunit 4	0.50 (−1.00)	NM_006693
NAP1L2	Nucleosome assembly protein 1-like 2	0.50 (−1.00)	NM_021963
LINC01501	Long intergenic non-protein coding RNA 1501	0.50 (−1.00)	NR_034157

Table S3. List of the enriched pathway based on Kyoto Encyclopedia of Genes and Genomes (KEGG) analysis of the differentially expressed genes from HEK293 cells following 5 μM PAT treatment for 10 h.

Gene symbol	Gene Name	Fold Change (log2)	transcriptID
	**Apoptosis**		
PSENEN	Presenilin enhancer gamma secretase subunit	4.26 (2.09)	NM_172341
BCL2L12	Bcl-2-like protein 12	3.85 (1.95)	NM_001040668
HMOX1	Heme oxygenase 1	3.20 (1.68)	NM_002133
CIAPIN1	Cytokin induced apoptosis inhibitor 1	2.68 (1.42)	NM_020313
RNF34	Ring finger protein 34, E3 ubiquitin protein ligase	2.58 (1.37)	NM_025126
DENND4B	DENN/MADD domain containing 4B	2.24 (1.16)	NM_014856
SLC7A5	Solute carrier family 7, member 5	2.06 (1.05)	NM_003486
AATF	Apoptosis antagonizing transcription factor	0.48 (−1.06)	NM_012138
CDKN2D	Cyclin-dependent kinase inhibitor 2D	0.46 (−1.13)	NM_079421
RHOC	Ras homology family member C	0.42 (−1.25)	NM_001042678
	**Oxidative phosphorylation**		
UQCR11	Cytochrome b-c1 complex subunit X1	2.47 (1.30)	NM_006830
NDUFA4	NADH dehydrogenase [ubiquinone] 1 alpha subcomplex subunit 4	2.45 (1.30)	NM_002489
ATP6V1C2	ATPase, H^+V^- transporting V1 subunit C 2	0.34 (−1.57)	NM_001039362
	**Oxidation-reduction process**		
ACADS	Acyl-CoA dehydrogenase	2.80 (1.48)	NM_000017
NDUFA4	NADH dehydrogenase [ubiquinone] 1 alpha subcomplex subunit 4	2.45 (1.30)	NM_002489
UQCR11	Cytochrome b-c1 complex subunit X1	2.47 (1.30)	NM_006830
	**Ribosome**		
RPS9	Ribosomal protein S9	3.72 (1.90)	NM_001013
CSTF3-AS1	CSTF3 antisense RNA1	3.31 (1.73)	NR_034027
LINC01623	Long intergenic non-protein coding RNA 1623	2.16 (1.11)	NR_033379
RPS20	Ribosomal protein S20	2.07 (1.05)	NM_001146227
RPLP2	Ribosomal protein, large, P2	2.02 (1.02)	NM_001004
RPL11	Ribosomal protein L11	0.39 (−1.35)	NM_001199802
	**RNA biosynthetic process**		
ARID3A	AT-rich interaction domain 3A	2.80 (1.48)	NM_005224
POLR1C	Polymerase (RNA) I polypeptide C	2.13 (1.09)	NM_203290
YBX3	Y-box binding protein 3	0.48 (-−1.05)	NM_003651
ZNF79	Zinc finger protein 79	0.48 (−1.06)	NM_007135
MZF1	Myeloid zinc finger 1	0.45 (−1.17)	NM_198055
MZF1-AS1	MZF1 anti sense RNA1	0.40 (−1.30)	NR_027334
	**RNA degradation**		
LSM1	LSM1 homology, mRNA degradation associated	3.01 (1.59)	NM_014462
PDE12	Phosphodiesterase 12	2.56 (1.34)	NM_177966
AMBRA1	Autophagy/beclin-1 regulator 1	0.45 (−1.14)	NM_017749
	**mRNA surveillance**		
RBM38	RNA binding motif protein 38	4.06 (2.02)	NM_183425
FIPL1	Factor interacting with PAPOLA and CPSF1	2.01 (1.14)	NM_001134937
CPSF4	Cleavage and polyadenylation specific factor 4	0.50 (−1.00)	NM_006693
	**RNA transport**		
TBC1D17	TBC1 domain family member 17	0.46 (−1.13)	NM_001168222
POP7	POP7 homology, ribonuclease P/MRP subunit	0.50 (−1.01)	NM_005837
	**DNA damage and repair**		
RBM38	RNA binding motif protein 38	4.06 (2.02)	NM_183425
FOXO4	Forkhead box O4	2.71 (1.44)	NM_005938
UIMC1	Ubiquitin interaction motif containing 1	2.44 (1.29)	NM_001199298
BRCA1	BRCA1 interacting protein C-terminal helicase 1	2.29 (1.20)	NM_032043

CDKN2D	Cyclin-dependent kinase inhibitor 2D(p19, inhibits CDK4)	0.46 (−1.13)	NM_079421
	**Cell cycle**		
RBM38	RNA-binding protein 38 isoform a	4.07 (2.02)	NM_183425
ARL8A	ADP-ribosylation factor-like protein 8A	3.73 (1.90)	NM_138795
UNKL	Unkempt-like RING finger protein	3.02 (1.60)	NM_023076
FOXO4	Forkhead box protein O4	2.71 (1.44)	NM_005938
HSPA8	Heat shock cognate 71 kDa protein	2.58 (1.37)	NM_006597
AATF	Apoptosis antagonizing transcription factor	0.48 (−1.06)	NM_012138
CDKN2D	Cyclin-dependent kinase inhibitor 2D (p19, inhibits CDK4)	0.46 (−1.13)	NM_079421
PKMYT1	Protein kinase, membrane associated tyrosine/threonine 1	0.45 (−1.10)	NM_182687
	**Cell growth**		
KAZALD1	Kazal-type serine peptidase inhibitor domain 1	455 (8.83)	NM_030929
CDKN2D	Cyclin-dependent kinase inhibitor 2D(p19, inhibits CDK4)	0.46 (−1.66)	NM_079421
RHOC	Ras homology family member C	0.42 (−1.26)	NM_001042678
CAPRIN2	Caprin family member 2	0.32 (−1.16)	NM_032156
	**Aging**		
DBAJA3	DnaJ heat shock protein family (HSP40) member A3	2.11 (1.08)	NM_001135110
HSD17B10	Hydroxysteroid (17-beta) dehydrogenase 10	2.08 (1.06)	NM_004493
	**Alzheimer's disease**		
PSENEN	Presenilin enhancer gamma secretase subunit	4.26 (2.09)	NM_172341
ATP6V1C2	ATPase, H+ transporting, lysosomal 42kDa, V1subunit C2	2.97 (1.57)	NM_001039362
UQCR11	Ubiquinol-cytochrome c reductase, complex III subunit XI	2.46 (1.30)	NM_006830
NDUFA4	NADH dehydrogenase (ubiquinone)1 alpha subcomples	2.01 (1.01)	NM_002489
HSD17B10	Hydroxysteroid (17-beta) dehydrogenase 10	2.08 (1.06)	NM_004493
	**Parkinson's disease**		
ATP6V1C2	ATPase, H+ transporting, lysosomal 42kDa, V1subunit C2	2.97 (1.57)	NM_001039362
UQCR11	Ubiquinol-cytochrome c reductase, complex III subunit XI	2.46 (1.30)	NM_006830
NDUFA4	NADH dehydrogenase (ubiquinone)1 alpha subcomples	2.01 (1.01)	NM_002489
**Cancer**			
CASC2	Cancer susceptibility 2 (non-protein coding)	0.44 (−1.17)	NR 026940
	**Prion disease**		
AJUBA	Ajuba LIM protein	3.02 (1.59)	NM_198086
NOTCH3	Notch 3	2.92 (1.55)	NM_000435
CAPRIN2	Caprin family member 2	0.32 (−1.66)	NM_032156
	**Type I diabetes mellitus**		
PDXDC1	Pyridocal-dependent decarboxylase domain containing 1	2.26 (1.18)	NM_015027
	**Amino acid metabolism**		
ACADS	Acyl-CoA dehydrogenase, C-2 to C-3 short chain	2.80 (1.48)	NM_000017
PDXDC1	Pyridocal-dependent decarboxylase domain containing 1	2.26 (1.18)	NM_015027
PNMAL1	Paraneoplastic Ma antigen family-like 1	2.22 (1.15)	NM_018215
HSD17B10	Hydroxysteroid (17-beta) dehydrogenase 10	2.08 (1.06)	NM_004493
	**Endocytosis**		
UNKL	Unkempt family zinc finger-like	3.02 (1.60)	NM_023076
GUCY1B3	Guanylate cyclase 1, soluble beta 3	2.67 (1.42)	NM_000857
UIMC1	Ubiquitin interaction motif containing 1	2.44 (1.29)	NM_001199298
SCARB1	Scavenger receptor class B member 1	2.34 (1.23)	NM_005505
CA7	Carbonic anhydrase VII	0.32 (−1.66)	NM_001014435
	**Neurotrophin signaling pathway**		
SHB	Src homology 2 domain containing adaptor protein B	0.47 (−1.08)	NM_003028
IRS4	Insulin receptor substrate 4	0.42 (−1.25)	NM_003604
	**NOD-Like receptor signaling pathway**		
AJUBA	Ajuba LIM protein	3.02 (1.59)	NM_198086
RNF34	Ring finger protein 34, E3 ubiquitin protein ligase	2.58 (1.37)	NM_025126
	**Phosphatidylinositol signaling system**		
PI4KA	Phosphatidylinositol 4-kinase	0.38 (−1.38)	NM_058004

When more than one assignment was available for a given gene, all the pathway annotations were considered in the analyses.

Table S4. Global changes of differentially expressed proteins (1.2-fold changes or greater, $P < 0.05$) quantified by iTRAQ from the cells following 5 μM PAT treatment for 10 h.

Gene name	Protein name	Score	Coverage (%)	No. of unique peptides	Bio 1	Bio 2	Mean
JUN	Jun proto-oncogen, transcription factor AP-1	172	8.8	2	2.327	2.259	2.293
SESN2	Sestrin-2	120	8.5	3	1.802	1.963	1.883
PFKFB3	6-phosphofructo-2-kinase 3	126	5.5	1	1.963	1.717	1.840
SLAIN2	SLAIN motif-containing protein 2	273	2.9	1	1.768	1.737	1.753
GMNN	Geminin	178	20.7	3	1.587	1.613	1.600
DMD	Dystrophin	84	2.2	2	1.824	1.361	1.593
DNPEP	Aspartyl aminopeptidase	73	5.3	2	1.793	1.363	1.578
DNAJB1	DNAJB1 protein	380	28.8	8	1.539	1.490	1.515
SLU7	Step II splicing factor SLU7		8.5	4	1.422	1.581	1.502
SPG20	Spastic paraplegia 20	194	7.7	3	1.414	1.554	1.484
MAP2K3	Dual-specificity mitogen-activated protein kinase kinase 3	104	30.9	3	1.463	1.505	1.484
CCRN4L	Nocturnin	151	3.5	1	1.549	1.412	1.481
BCL10	B-cell lymphoma/leukemia 10	103	17.3	3	1.394	1.566	1.480
BCL7C	B-cell CLL/lymphoma 7 protein family member C	92	7.5	1	1.46	1.499	1.4795
CCNA2	Cyclin-A2	161	7.2	2	1.304	1.654	1.479
RBM33	RNA-binding protein 33	92	2.4	2	1.502	1.451	1.477
CASP3	Caspase-3	178	11.2	4	1.346	1.597	1.472
PHF5A	PHD finger-like domain-containing protein 5A	258	31.8	4	1.507	1.424	1.466
YY1	Transcriptional repressor protein YY1	144	7	3	1.376	1.542	1.459
PI5K1A	Phosphatidylinositol 4-phosphate 5-kinase type-1 alpha	216	10.9	1	1.335	1.559	1.447
NFKBIB	NF-kappa-B inhibitor beta	75	8.7	1	1.391	1.493	1.442
TUBAL3	Tubulin alpha chain-like 3	283	8.7	1	1.303	1.554	1.429
NUSAP1	Nucleolar and spindle associated protein 1	129	15.6	6	1.49	1.306	1.398
LUZP1	Leucine zipper protein 1	139	5.9	5	1.329	1.451	1.390
ARL5B	ADP-ribosylation factor-like protein 5B	100	14	2	1.447	1.328	1.388
CDC42EP4	Cdc42 effector protein 4	190	19.1	4	1.307	1.444	1.376
PPP1R2	Proteinphosphatase inhibitor 2-like	364	16.6	2	1.465	1.256	1.361
GCLM	Glutamate--cysteine ligase regulatory subunit	155	20.1	4	1.416	1.279	1.348
PSME2	Proteasome activator complex subunit 2	256	16.9	4	1.308	1.386	1.347
KDM2A	Lysine-specific demethylase 2A	122	5.2	5	1.420	1.267	1.344
MYO1E	Unconventional myosin-Ie	161	6.2	5	1.318	1.349	1.334
NHERF	Na(+)/H(+) exchange regulatory cofactor	511	17.3	4	1.361	1.290	1.326
PARD6B	Partitioning defective 6 homolog beta	76	3.8	1	1.265	1.383	1.324
TAF4	Transcription initiation factor TFIID subunit 4	117	4.1	3	1.286	1.351	1.319
CCNH	Cyclin-H	78	10.2	2	1.318	1.307	1.313
CBFB	Core-binding factor subunit beta	800	25.3	4	1.301	1.316	1.309
FRG1	Protein FRG1	93	32.8	3	1.220	1.397	1.309
CBFB	Core-binding factor subunit beta	800	25.3	4	1.301	1.316	1.309
KLP	Kinesin-like protein	443	13.5	8	1.220	1.351	1.286
ZNHIT6	Box C/D snoRNA protein 1	96	8.7	3	1.304	1.263	1.284
ACYP1	Acylphosphatase	220	40.3	6	1.351	1.208	1.280
ANLN	Actin-binding protein anillin	111	6.6	6	1.294	1.246	1.270
TBCB	Tubulin-folding cofactor B	162	24.1	4	1.223	1.305	1.264
TACC1	Transforming acidic coiled-coil-containing protein 1	133	4	2	1.215	1.309	1.262
KIFAP3	Kinesin-associated protein 3	50	3.2	2	1.206	1.283	1.245
PDCD4	Programmed cell death 4	136	9	4	1.222	1.250	1.236
SLC3A2	4F2 cell-surface antigen heavy chain	765	25	13	1.245	1.204	1.225
EP300	Histone acetyltransferase p300	114	3.2	1	1.207	1.220	1.214
INCENP	Inner centromere protein	132	3.5	2	1.204	1.200	1.202

NKTR	NK-tumor recognition protein	132	3.4	3	0.826	0.826	0.826
POLDIP2	Polymerase delta-interacting protein 2	694	30.7	10	0.816	0.819	0.818
RPS13	40S ribosomal protein S13	419	46.4	8	0.826	0.805	0.816
ACADSB	Short/branched chain specific acyl-CoA dehydrogenase, mitochondrial	174	19.7	6	0.817	0.808	0.813
ATP6	ATP synthase subunit a	235	4.4	1	0.821	0.773	0.797
NDC1	Nucleoporin NDC1	171	8.6	6	0.824	0.764	0.794
ATP5O	ATP synthase subunit O, mitochondrial	686	57.7	9	0.812	0.774	0.793
MRPL19	Mitochondrial ribosomal protein L19	139	16.8	6	0.753	0.830	0.792
FIS1	Mitochondrial fission 1 protein	130	7.2	1	0.757	0.823	0.790
PUF60	Poly(U)-binding-splicing factor PUF60	1149	34.3	5	0.818	0.760	0.789
HADH	Hydroxyacyl-coenzyme A dehydrogenase	220	14.9	6	0.806	0.767	0.787
MRPL3	39S ribosomal protein L3, mitochondrial	118	6.3	3	0.826	0.735	0.781
RBM1	RNA binding motif protein, family 1	1321	20.3	1	0.759	0.800	0.780
MRPL54	39S ribosomal protein L54, mitochondrial	105	7.2	1	0.738	0.816	0.777
MRPL11	39S ribosomal protein L11, mitochondrial	590	51	9	0.791	0.759	0.775
RPS17L	40S ribosomal protein S17-like	456	55.6	6	0.781	0.768	0.775
RPL35	Ribosomal protein L35	172	10.1	1	0.789	0.753	0.771
BANF1	Barrier-to-autointegration factor	540	40.4	2	0.750	0.783	0.767
SLC39A14	Zinc transporter ZIP14 (Fragment)	124	28.1	2	0.826	0.692	0.759
NCSTN	Nicastrin	219	6.8	5	0.770	0.7407	0.759
FXN	Frataxin, mitochondrial	141	22.2	2	0.764	0.752	0.758
NOL7	Nucleolar protein 7	186	15.2	4	0.769	0.739	0.754
RDH13	Retinol dehydrogenase 13	82	11.8	4	0.820	0.670	0.745
H1F0	Histone H1.0	326	16.5	4	0.81	0.679	0.745
NDUFAF4	NADH dehydrogenase [ubiquinone] 1 alpha subcomplex assembly factor 4	199	32.6	6	0.783	0.691	0.737
MRPS7	28S ribosomal protein S7, mitochondrial	653	30.6	7	0.830	0.640	0.735
PABPN1	Polyadenylate-binding protein 2	145	28.1	4	0.687	0.772	0.730
CPD	Carboxypeptidase D	283	8.5	10	0.795	0.655	0.725
MRPS10	Mitochondrial 28S ribosomal protein S10	100	18.8	2	0.736	0.696	0.716
GTPBP3	GTPBP3 protein (Fragment)	54	4.9	2	0.628	0.799	0.714
LYRM7	Complex III assembly factor LYRM7	97	15.4	2	0.748	0.669	0.709
FAU	40S ribosomal protein S30	95	11.2	2	0.759	0.641	0.700
ERAL1	GTPase Era, mitochondrial	160	15.6	5	0.712	0.678	0.695
NDUFA5	NADH dehydrogenase [ubiquinone] 1 alpha subcomplex subunit 5	334	24.8	2	0.759	0.616	0.688
NDUFA6	NADH dehydrogenase [ubiquinone] 1 alpha subcomplex subunit 6	145	11.3	1	0.690	0.603	0.647

Bio1 represent 113:115, while bio2 represent 114:116.

Table S5. List of the enriched pathway based on KEGG analysis of the differentially expressed proteins from HEK293 cells following 5 μM PAT treatment for 10 h.

Gene name	Protein name	Score	Coverage (%)	No. of unique peptides	Bio 1	Bio 2	Mean
**Apoptosis**							
JUN	Transcription factor AP-1	172	8.8	2	2.327	2.259	2.293
SPG20	Spastic paraplegia 20, spartin	194	7.7	3	1.414	1.554	1.484
BCL10	B-cell lymphoma/leukemia 10	103	17.3	3	1.394	1.566	1.480
BCL7C	B-cell CLL/lymphoma 7 protein family member C	92	7.5	1	1.460	1.499	1.480
CCNA2	Cyclin-A2	161	7.2	2	1.304	1.654	1.479
CASP3	Caspase-3	178	11.2	4	1.346	1.597	1.472
NFKBIB	NF-kappa-B inhibitor beta	75	8.7	1	1.391	1.493	1.442
BUB1	BUB1 protein	167	5.3	3	1.262	1.312	1.287
PDCD4	Programmed cell death 4 (neoplastic transformation inhibitor)	136	9	4	1.222	1.250	1.236
EP300	Histone acetyltransferase p300	114	3.2	1	1.207	1.220	1.214
H1F0	Histone H1.0	326	16.5	4	0.810	0.679	0.745
**Oxidative phosphorylation**							
ATP6	ATP synthase subunit a	235	4.4	1	0.821	0.773	0.797
ATP5O	ATP synthase subunit O, mitochondrial	132	3.4	3	0.812	0.774	0.793
NDUFAF4	NADH dehydrogenase [ubiquinone] 1 alpha subcomplex assembly factor 4	199	32.6	6	0.783	0.691	0.737
NDUFA5	NADH dehydrogenase [ubiquinone] 1 alpha subcomplex subunit 5	334	24.8	2	0.759	0.616	0.688
NDUFA6	NADH dehydrogenase [ubiquinone] 1 alpha subcomplex subunit 6	145	11.3	1	0.690	0.603	0.647
**Oxidation-reduction process**							
PPP1R2	Protein phosphatase inhibitor 2-like protein	364	16.6	2	1.465	1.256	1.361
ACADSB	Short/branched chain specific acyl-CoA dehydrogenase, mitochondrial	174	19.7	6	0.817	0.808	0.813
HADH	Hydroxyacyl-coenzyme A dehydrogenase	220	14.9	6	0.806	0.767	0.787
NDUFA5	NADH dehydrogenase [ubiquinone] 1 alpha subcomplex subunit 5	334	24.8	2	0.759	0.616	0.688
NDUFA6	NADH dehydrogenase [ubiquinone] 1 alpha subcomplex subunit 6	145	11.3	1	0.690	0.603	0.647
**Ribosomal**							
RPS13	40S ribosomal protein S13	419	46.4	8	0.826	0.805	0.816
MRPL19	Mitochondrial ribosomal protein L19	139	16.8	6	0.753	0.830	0.792
MRPL3	39S ribosomal protein L3, mitochondrial	118	6.3	3	0.826	0.735	0.781
MRPL54	39S ribosomal protein L54, mitochondrial	105	7.2	1	0.738	0.816	0.777
MRPL11	39S ribosomal protein L11, mitochondrial	590	51	9	0.791	0.759	0.775
RPS17L	40S ribosomal protein S17-like	456	55.6	6	0.781	0.768	0.775
RPL35	Ribosomal protein L35	172	10.1	1	0.789	0.753	0.771
MRPS7	28S ribosomal protein S7, mitochondrial	653	30.6	7	0.830	0.640	0.735
MRPS10	Mitochondrial 28S ribosomal protein S10	100	18.8	2	0.736	0.696	0.716
FAU	40S ribosomal protein S30	95	11.2	2	0.759	0.641	0.700
**RNA biosynthetic process**							
NOCT	Nocturnin	151	3.5	1	1.549	1.412	1.481
YY1	Transcriptional repressor protein YY1	144	7	3	1.376	1.542	1.459
TAF4	Transcription initiation factor TFIID subunit 4	117	4.1	3	1.286	1.351	1.319
CCNH	Cyclin-H	78	10.2	2	1.318	1.307	1.313
SRSF6	Arginine/serine-rich splicing factor 6 variant	155	17.2	3	0.779	0.810	0.795
PUF60	Poly(U)-binding-splicing factor	1149	34.3	5	0.818	0.760	0.789
PABPN1	Polyadenylate-binding protein 2	145	28.1	4	0.687	0.772	0.730
**RNA degradation**							
NOCT	Nocturnin	151	3.5	1	1.549	1.412	1.481
**mRNA surveillance pathway**							
PABPN1	Polyadenylate-binding protein 2	145	28.1	4	0.687	0.772	0.730
**DNA damage**							

CCNA2	Cyclin-A2	161	7.2	2	1.304	1.654	1.479
PSME2	Proteasome activator complex subunit 2	256	16.9	4	1.308	1.386	1.347
**Proteasome**							
PSME2	Proteasome activator complex subunit 2	256	16.9	4	1.308	1.386	1.347
**Cell cycle**							
SESN2	Sestrin-2	120	8.5	3	1.802	1.963	1.883
CCNA2	Cyclin-A2	161	7.2	2	1.304	1.654	1.479
NUSAP1	Nuclear and spindle associated protein 1	129	15.6	6	1.490	1.306	1.398
PARD6B	Partitioning defective 6 homolog beta	76	3.8	1	1.265	1.383	1.324
CCNH	Cyclin-H	78	10.2	2	1.318	1.307	1.313
ANLN	Actin-binding protein anillin	111	6.6	6	1.294	1.246	1.270
TACC1	Transforming acidic coiled-coil-containing protein 1	133	4	2	1.215	1.309	1.262
**Endocytosis**							
PIP5K1A	Phosphatidylinositol 4-phosphate 5-kinase type-1 alpha	216	10.9	1	1.335	1.559	1.447
PARD6B	Partitioning defective 6 homolog beta	76	3.8	1	1.265	1.383	1.324
PPP1RSP3	Protein phosphatase inhibitor 2-like	364	16.6	2	1.465	1.256	1.361
**Alzheimer′s disease**							
CASP3	Caspase-3	178	11.2	4	1.346	1.597	1.472
ATP6	ATP synthase subunit a	235	4.4	1	0.821	0.773	0.797
ATP5O	ATP synthase subunit O, mitochondrial	132	3.4	3	0.812	0.774	0.793
NCSTN	Nicastrin	219	6.8	5	0.770	0.747	0.759
NDUFA5	NADH dehydrogenase [ubiquinone] 1 alpha subcomplex subunit 5	334	24.8	2	0.759	0.616	0.688
NDUFA6	NADH dehydrogenase [ubiquinone] 1 alpha subcomplex subunit 6	145	11.3	1	0.690	0.603	0.647
**Parkinson′s disease**							
CASP3	Caspase-3	178	11.2	4	1.346	1.597	1.472
TAF4	Transcription initiation factor TFIID subunit 4	117	4.1	3	1.286	1.351	1.319
EP300	Histone acetyltransferase p300	114	3.2	1	1.207	1.220	1.214
ATP6	ATP synthase subunit a	235	4.4	1	0.821	0.773	0.797
ATP5O	ATP synthase subunit O, mitochondrial	132	3.4	3	0.812	0.774	0.793
NDUFA5	NADH dehydrogenase [ubiquinone] 1 alpha subcomplex subunit 5	334	24.8	2	0.759	0.616	0.688
NDUFA6	NADH dehydrogenase [ubiquinone] 1 alpha subcomplex subunit 6	145	11.3	1	0.690	0.603	0.647
**Pathways in cancer**							
JUN	Transcription factor AP-1	172	8.8	2	2.327	2.259	2.293
CCNA2	Cyclin-A2	161	7.2	2	1.304	1.654	1.479
CASP3	Caspase-3	178	11.2	4	1.346	1.597	1.472
EP300	Histone acetyltransferase p300	114	3.2	1	1.207	1.220	1.214
**Amino acid metabolism**							
BCL7C	B-cell CLL/lymphoma 7 protein family member C	92	7.5	1	1.460	1.499	1.480
GCLM	Glutamate-cysteine ligase regulatory subunit	155	20.1	4	1.416	1.279	1.348
ACADSB	Short/branched chain specific acyl-CoA dehydrogenase, mitochondrial	174	19.7	6	0.817	0.808	0.813
HADA	Hydroxyacyl-coenzyme A dehydrogenase, mitochondrial	220	14.9	6	0.806	0.767	0.787

When more than one assignment was available for a given protein, all the pathway annotations were considered in the analyses.

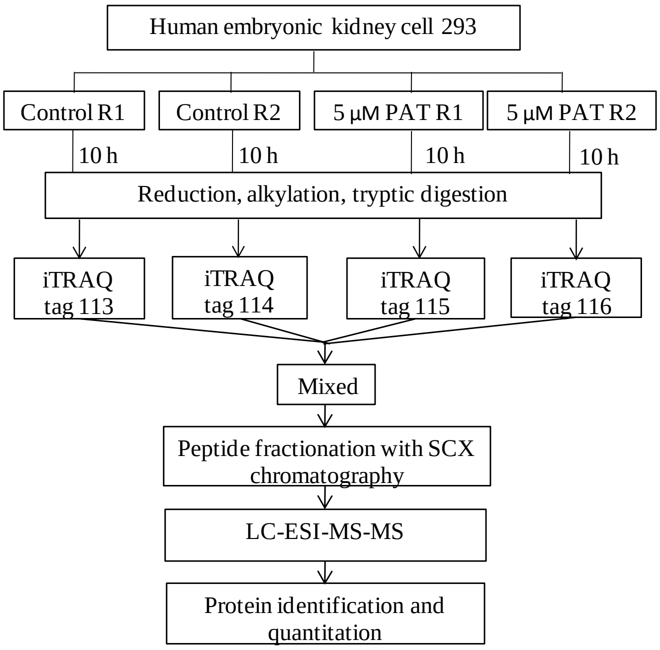

Figure S2. Experimental design for iTRAQ labeling to explore the effect of 5 μM PAT on HEK293 cells. R1 represent repeat 1, R2 represent repeat 2.

Table S6. Gene specific primers used in real-time quantitative PCR.

Gene name	Forward primer	Reverse primer	Product bp
HSPB8	aaagatggatacgtggaggt	caaatgttgagtaaggaggga	190
SLC25A6	tcattcgctacttccccact	ctctgtgcctgactttccca	221
RNF34	gctgtgactgcaagaaggat	gcgctgaaatgctgtctctt	190
ASL	actgcaccaaggaattcagc	tgttgtaggtgctgggaagt	180
COX17	tagatttggctgtctccgct	ctctcatgcattccttgtggg	213
FIS1	aaagggagcaaggaggaaca	atgcccacgagtccatcttt	188
